# Hemodynamic response varies across tactile stimuli with different temporal structures

**DOI:** 10.1002/hbm.25243

**Published:** 2020-11-10

**Authors:** Luyao Wang, Chunlin Li, Duanduan Chen, Xiaoyu Lv, Ritsu Go, Jinglong Wu, Tianyi Yan

**Affiliations:** ^1^ School of Mechatronical Engineering Beijing Institute of Technology Beijing China; ^2^ School of Biomedical Engineering Capital Medical University Beijing China; ^3^ School of Life Science Beijing Institute of Technology Beijing China; ^4^ Beijing Advanced Innovation Center for Intelligent Robots and Systems Beijing Institute of Technology Beijing China; ^5^ Graduate School of Interdisciplinary Science and Engineering in Health Systems Okayama University Okayama Japan

**Keywords:** duration, frequency, hemodynamic response, tactile stimulus, temporal structure

## Abstract

Tactile stimuli can be distinguished based on their temporal features (e.g., duration, local frequency, and number of pulses), which are fundamental for vibrotactile frequency perception. Characterizing how the hemodynamic response changes in shape across experimental conditions is important for designing and interpreting fMRI studies on tactile information processing. In this study, we focused on periodic tactile stimuli with different temporal structures and explored the hemodynamic response function (HRF) induced by these stimuli. We found that HRFs were stimulus‐dependent in tactile‐related brain areas. Continuous stimuli induced a greater area of activation and a stronger and narrower hemodynamic response than intermittent stimuli with the same duration. The magnitude of the HRF increased with increasing stimulus duration. By normalizing the characteristics into topographic matrix, nonlinearity was obvious. These results suggested that stimulation patterns and duration within a cycle may be key characters for distinguishing different stimuli. We conclude that different temporal structures of tactile stimuli induced different HRFs, which are essential for vibrotactile perception and should be considered in fMRI experimental designs and analyses.

## Graphical abstract

We found that HRFs were stimulus‐dependent in tactile‐related brain areas. Stimulation patterns and duration within a cycle may be key characters for distinguishing different stimuli.

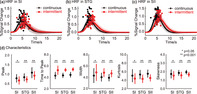

## 1 INTRODUCTION

Blood oxygen level‐dependent (BOLD) signals measured by functional magnetic resonance imaging (fMRI) could be used to indirectly infer neural activity. The waveform of an assumed hemodynamic response function (HRF) plays a key role in fMRI analysis, which is regarded as the vascular response evoked by a brief neural event (Boynton, Engel, Glover, & Heeger, 1996). Studies found it varies in shape across cortical regions (Handwerker, Ollinger, & D'Esposito, 2004; Puckett, Mathis, & Deyoe, 2014) and ages (Liu, Gerraty, Grinband, Parker, & Razlighi, 2017), which potentially reflect different local distributions of vascular anatomy and morphology. Incorrect assumptions about HRFs can lead to substantial inference errors (Greve, Brown, Mueller, Glover, & Liu, 2013; Liu, Duffy, Bernal‐Casas, Fang, & Lee, 2017). Furthermore, there has been increasing interest in characterizing the peak amplitude, onset, and duration of evoked hemodynamic responses across experimental conditions, which may be used to infer information about intensity, onset latency, and duration of the underlying neuronal activity (Lindquist, Loh, Atlas, & Wager, 2009). Studies using visual stimuli of varying durations found HRFs exhibit nonlinearities (Lewis, Setsompop, Rosen, & Polimeni, 2018). Shorter duration stimuli can elicit larger and faster responses than would have been predicted by a linear model. This means the hemodynamic response is also stimulus dependent. Exploring the hemodynamic response properties in different experimental conditions will be of increasing importance when exploring the process of how humans perceive stimuli.

To date, most studies focused on estimating HRFs across visual cortex (Puckett et al., 2014), frontal areas (Handwerker et al., 2004; Taylor, Kim, & Ress, 2018), and subcortical areas (Lewis et al., 2018). To our knowledge, few studies explored the properties of the hemodynamic response induced by tactile stimuli. Touch has traditionally been considered a spatial sense. Researchers have often studied brain activity using stimulus with different spatial configurations, such as somatotopic maps (Sanchez‐Panchuelo et al., 2014; Sanchez‐Panchuelo, Francis, Bowtell, & Schluppeck, 2010). However, there are aspects of touch that are unrelated to the spatial dimension, in particular its temporal precision and the putative functional role thereof. It was reported that BOLD activation exponentially decreased due to long‐term tactile stimuli (up to 15 s) (Chung et al., 2015). Studies on macaque monkeys shown that tactile stimuli can be distinguished based on their temporal features, which are fundamental for vibrotactile frequency perception (Harvey, Saal, Dammann, & Bensmaia, 2013; Mackevicius, Best, Saal, & Bensmaia, 2012). The temporal structures of tactile stimuli are important components in human judgments of surface texture and object recognition (Yang et al., 2017; Yu, Yang, Ejima, Fukuyama, & Wu, 2017). Human behavioral studies found tactile frequency perception was similar to auditory frequency perception, in which the duration of silent gap between successive bursts was more important than underlying periodicity (Birznieks & Vickery, 2017). In clinical interventions, tactile stimulation can enhance sensorimotor function, and the results greatly varied across different parameters, such as duration, frequency and amplitude (Lesemann, Reuter, & Godde, 2015). However, how peripheral tactile stimulation translates into perception at the cortical level is still unknown.

Tactile information is transmitted from peripheral receptors to the cuneate, thalamus, primary somatosensory cortex (SI) and secondary somatosensory cortex (SII) (Abraira & Ginty, 2013). Along this path, thalamic and SI neurons mostly encode the physical features of the stimulus, such as location, duration and frequency (Sherman, 2016). In addition, sounds and vibrations may not be independently represented and processed so that they could evoke responses in the same area (Kayser, Petkov, Augath, & Logothetis, 2005; Nordmark, Pruszynski, & Johansson, 2012; Schurmann, Caetano, Hlushchuk, Jousmaki, & Hari, 2006). Recent studies reported that tactile stimulation could modulate activity in the temporal lobes (Perez‐Bellido, Barnes, Crommett, & Yau, 2018). In this study, we focused on the basic tactile processing circuits, including the SI, SII, and temporal lobe, and explored the hemodynamic response induced by tactile stimuli of different frequencies and durations. We used periodic stimuli (1 Hz) of the same intensity, in which local frequencies were different (continuous and intermittent stimuli). We hypothesize that the HRFs in all regions of interest (ROIs) induced by two types of tactile stimuli have different characteristics. The nonlinearities also exist in tactile regions. In addition, duration maybe important component within a stimulus cycle. Our findings will highlight the temporal features of a tactile stimulus that should be considered when analyzing fMRI data.

## 2 MATERIALS AND METHODS

### 2.1 Participants

Twenty healthy participants participated in the experiments (7 females; age range: 21–30 years old; mean age: 24.7 years old). Another eight healthy participants performed the experiment at a higher resolution to verify the results. All of them were right‐handed according to the Edinburgh Handedness Inventory (Oldfield, 1971). The protocol and data collection of the study were approved by the ethics committee of Capital Medical University in accordance with the declaration of Helsinki. All participants provided written informed consent.

### 2.2 Stimulus and tasks

**FIGURE 1:**
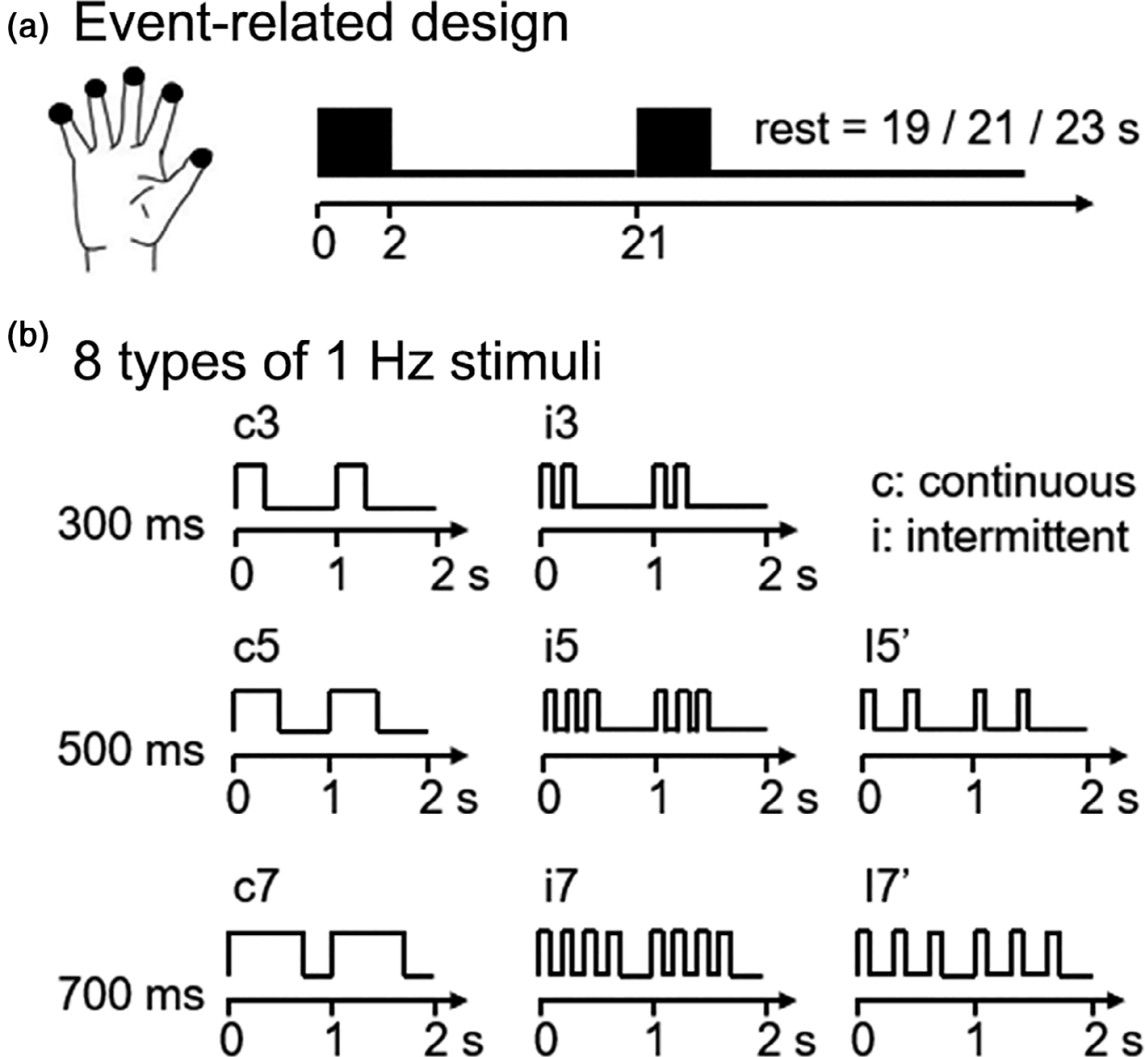
Experimental paradigm of event‐related design. (a) Example of two event trails, which used 2‐s stimulus presentation and random rest intervals (19–23 s). (b) Eight types of 1 Hz stimuli. The naming rules are as follows: the first letter represents the pattern of the stimulus (c, continuous; i, intermittent), and the digit represents the duration of the stimulus

Tactile stimuli were presented using a pneumatic air‐jet stimulator system, which could provide steady stimulus to fingers (Jia et al., 2020). Five finger tips of the right hand were stimulated simultaneously in the context of event‐related paradigm (Figure 1a). The pressure of tactile stimuli applied to the hand was controlled at 150 mN by adjusting the input air pressure. To control the attentional state, the experiment was divided into eight runs. Participants were asked to count the number of trials in each run, in which comprised 29–31 stimulus trials with 2‐s periods of stimulation and a random interstimulus intervals (ISI) ranging from 19 to 23 s (average is 21 s). In this manner, expectations could also be avoided (Liu, Gerraty, Grinband, Parker, & Razlighi, 2017).

Eight types of stimuli were designed with an overall frequency of 1 Hz (Figure 1b). In addition, they were divided into two patterns. The first pattern was continuous stimulus (c) with three durations (c3: 300 ms, c5: 500 ms, and c7: 700 ms). The second pattern was intermittent stimulus (i) with a local frequency of 5 Hz and durations matching the first group (i3, i5, and i7). Then, we added two supplemental conditions of intermittent stimulation to further explore the influence of pulses number within one period to the hemodynamic response. The first condition was stimulation with two pluses for 500 ms duration (i5′), and the second condition was stimulation with three pulse for 700 ms duration (i7′).

### 2.3 Magnetic resonance imaging data acquisition and preprocessing

The fMRI data were acquired using a 3.0‐Tesla Siemens MAGNETOM Prisma scanner to measure activation with a 64‐channel head coil. An interleaved T2*‐weighted gradient‐echo planar imaging (EPI) scan was performed to acquire functional images covering the entire brain (repetition time (TR) = 2000 ms, echo time (TE) = 30 ms, flip angle = 90°, matrix = 64 × 64, voxel size = 3.5 × 3.5 × 4.2 mm, slice gap = 0 mm, 33 axial slices per volume, GRAPPA factor = 2). In verification experiment, we used finer spatial resolution with matrix 64 × 64, 2 × 2 × 2.5 mm voxel size, 25 coronal slices per volume, and other parameters were same as main dataset. The phase‐encode direction was set to left–right to avoid wrap‐around. T1‐weighted magnetization‐prepared rapid acquisition gradient‐echo (MP‐RAGE) fine structural images of the entire head were also acquired (TR = 2,300 ms, TE = 2.36 ms, TI = 900 ms, flip angle = 8°, FOV = 256 × 256 mm, voxel size = 0.84 × 0.84 × 1.0 mm).

Preprocessing steps were carried out using the SPM12 software (Wellcome Department of Cognitive Neurology, London, UK). The processing of the fMRI data included slice timing correction, spatial realignment, coregistration, normalization to Montreal Neurological Institute (MNI) space based on the average bold image resliced in realignment, and smoothing with an isotropic Gaussian kernel of 8 mm full‐width at half‐maximum (FWHM) for main dataset. There was no smooth for verification data set. A general linear model (GLM) analysis was carried out to estimate the activation induced by stimulus, which included six head motion parameters as nuisance regressors.

### 2.4 Dissimilarity analysis

The goal of the dissimilarity analysis was to investigate whether multi‐voxel patterns of activations associated with the stimulus types were different. After GLM analysis, we extracted all estimated beta parameters in each anatomical ROI. The stereotypical anatomical locations of the SI (BA1, 2, and 3), superior temporal gyrus (STG), and SII were identified using the automated anatomical labeling (AAL) template. SII spherical ROIs with coordinates of (−53, −26, 20) and (53, −28, 22) in MNI space with a radius of 15 mm were defined based on a meta‐analysis of all noxious stimuli (Duerden & Albanese, 2013). All the anatomical ROIs were defined using WFU PickAtlas 3.0.5 (Maldjian, Laurienti, Kraft, & Burdette, 2003). The representational dissimilarity matrix (RDM) between each pair of stimulus types was calculated using correlation distance based on beta values. Then, the RDM was averaged in each ROI and statistically analyzed using paired *t* test to test differences across brain areas.

### 2.5 Analysis of peak activation and extent areas

We investigated the coordinates of the peak activation voxel in anatomical ROIs to explore whether different types of stimulus could induce different spatial configurations (*p* < .001, cluster corrected). In addition, because the total volume across anatomical ROIs was different, we analyzed the extent area using the relative volume of activation, not the absolute volume (activation volume). The number of activated voxels within each ROI were normalized by the total number of voxels of the corresponding ROI (Akselrod et al., 2017). This method measured the fraction occupied by a given stimulus type to account for volumetric differences. In addition, based on a conjunction analysis, subregions that were tactilely driven by our stimulus were further constrained using a spatial extent representing the intersection of all task conditions (*p* < .001, cluster corrected). These functional ROIs were used for further analysis including only voxels that showed significant responses to the task in all conditions.

### 2.6 HRF deconvolution

A deconvolution analysis was performed between the full time‐course of each voxel and the stimulus timing waveform. We used both unsmoothed and smoothed data to estimate HRF induced by eight stimuli. The HRF was modeled by the sum of two gamma functions:

(1) $$h\left(t\right)=A\left(\frac{t^{\alpha_{1}-1}\beta_{1}^{\alpha_{1}}e^{-\beta_{1}t}}{\Gamma \left(\alpha_{1}\right)}-c\frac{t^{\alpha_{2}-1}\beta_{2}^{\alpha_{2}}e^{-\beta_{2}t}}{\Gamma \left(\alpha_{2}\right)}\right)$$

Six free parameters are assumed to be unknown, where A represents the amplitude, αand βrepresent the shape and scale, respectively, and c determines the ratio of the response to undershoot. An unconstrained nonlinear search algorithm was used to identify the best fitting parameters (MATLAB's *fminsearch* function) (Handwerker et al., 2004; Lindquist et al., 2009; Liu, Duffy, et al., 2017; Shan et al., 2014). To assess the capability of estimation, the root‐mean‐square error (RMSE) was calculated relative to the observed fMRI time‐course.

The mean signal value during the first rest period was calculated as the baseline value, and then each voxel within ROIs was estimated individually. The percentage BOLD signal change at each time point was calculated based on the baseline value (Shan et al., 2014; Wen, Liu, Yao, & Ding, 2013). Then, the averaged hemodynamic responses and characteristics were used for statistical analysis across stimulus types. We used the following summary HRF characteristics: peak, defined as the maximum signal change during the stimulus time window (30 s was used for this study); peak time, the time taken from start time to the time when the signal change reached its maximum value; FWHM; kurtosis and skewness, which represent asymmetry or higher‐order statistics of HRF. All processes were performed using an inhouse MATLAB program.

Previous studies have shown that the hemodynamic response exhibits nonlinearities across the visual cortex. The nonlinearity is represented by the shape of the HRF changing as the stimulus changes. These changes are mainly reflected in the dynamics and amplitude of the HRF. To explore nonlinearity in the tactile cortex, the characteristics of the HRF were normalized by the stimulus duration and the number of pulses within 1 s period (Lewis et al., 2018). If the normalized index falls on the line y = 1, the hemodynamic response changes linearly with the stimulus; otherwise, a nonlinear response exists. The degree of nonlinearity was computed as the best‐fit straight line to the normalized index (i.e., steep negative slope = highly nonlinear, slope of 0 = linear). To obtain confidence intervals and perform statistical testing, 1,000 times bootstrap resampling was conducted over subject. In addition, to explore the variation across characteristics, stimulus duration, and number of pulses, characteristics were fitted to topographic maps.

### 2.7 Statistical analysis

SPSS version 23 (SPSS, Inc., Chicago, IL) was used for statistical analysis. A paired‐sample *t* test was performed to test averaged RDM across each ROI (1,000 times bootstrap resampling was conducted over subjects). In addition, repeated measures ANOVA were performed when comparing extent areas and characteristics of HRF. Post hoc Bonferroni‐corrected pair‐wise comparisons were used to assess differences between levels of significant factors. The significance level was set to α = 0.05 for both ANOVAs and post hoc tests. We also assessed the sphericity and normality of the data. In case of violation of sphericity, we conducted a Greenhouse–Geisser correction on the ANOVA (Geisser & Greenhouse, 1958).

## 3 RESULTS

### 3.1 Different spatial activation configurations

**FIGURE 2:**
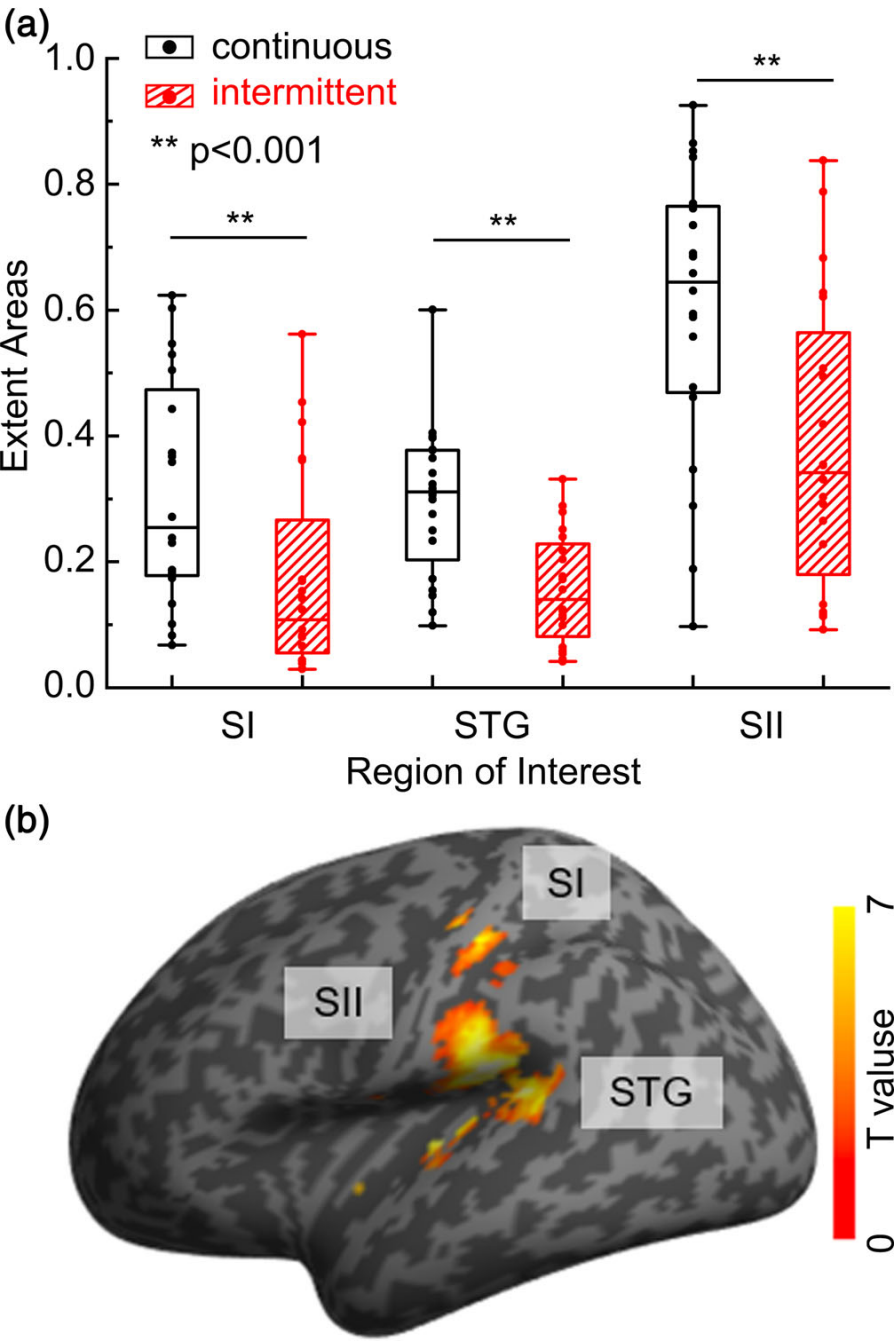
Extent areas of the two patterns of stimuli and region of interest (ROI). (a) The vertical axis represents the relative volume of activation in each anatomical ROI. Black boxes are the extent areas for the continuous stimuli, and red boxes are for the intermittent stimuli. (b) Functional ROIs used in following analysis based on conjunction analysis, which include primary somatosensory cortex (SI), secondary somatosensory cortex (SII), and superior temporal gyrus (STG)

We recorded the spatial coordinates of the peak activation voxel (Table S1). There was no sequential arrangement across different stimulus types. For stimulus c3, c5, c7, i3, i5, and i7, the extent areas were also measured and compared by a two‐way repeated measures analysis of variance (ANOVA) with pattern (2 levels) and duration (3 levels) as within‐subject factors. The comparison was performed separately for each ROI. A significant main effect of pattern was found in all three ROIs (SI: *p* < .001, F (1, 19) = 37.768; STG: *p* < .001, F (1, 19) = 54.013; SII: *p* < .001, F (1, 19) = 30.175). This result suggested that the continuous stimuli significantly induced larger extent than the intermittent stimulus (Figure 2a). However, there was no significant main effect of duration.

### 3.2 Dissimilarity analysis across areas

**FIGURE 3:**
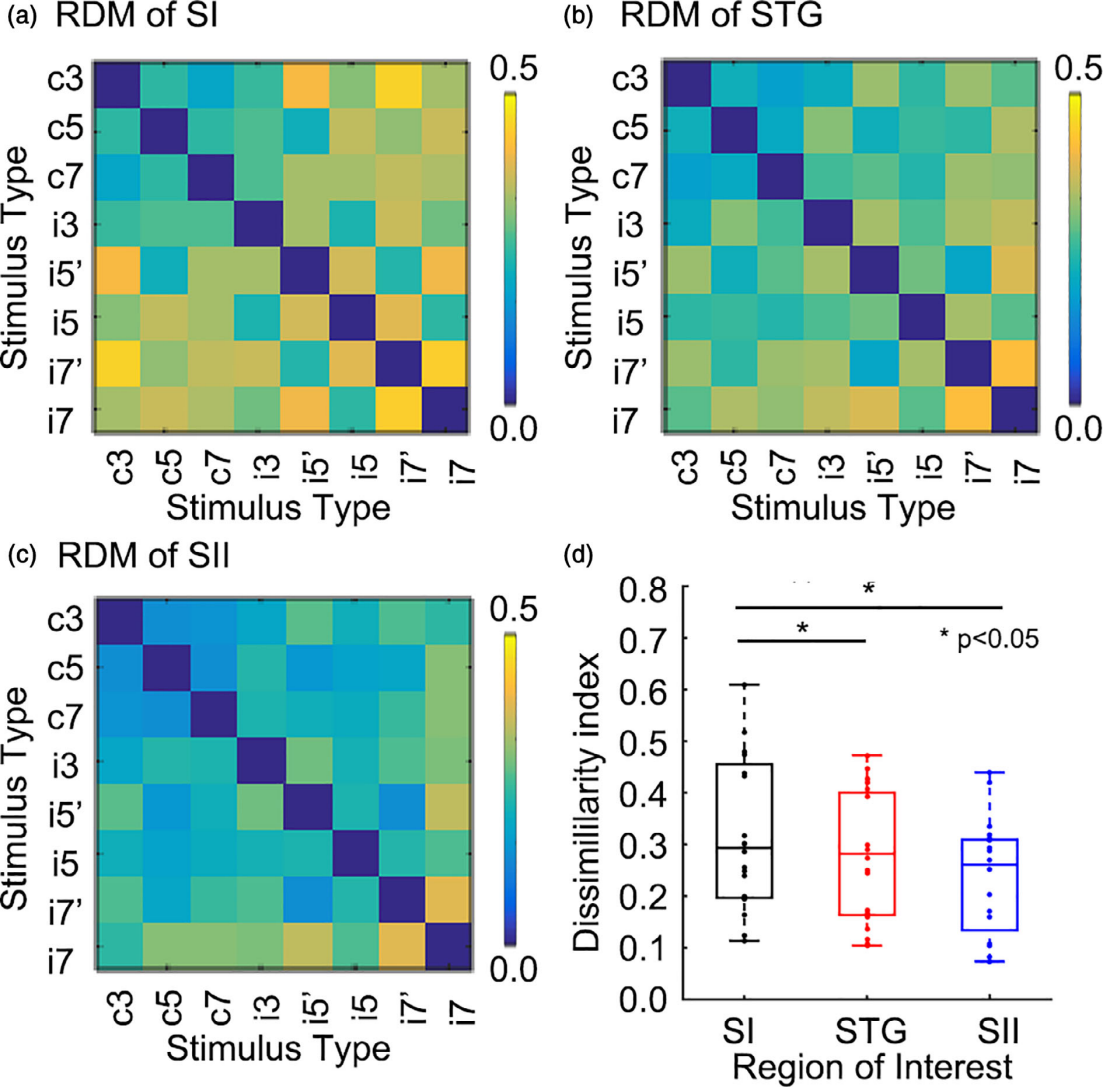
Results of the dissimilarity analysis in different anatomical regions of interest. (a) RDM result of all voxels in SI. The value between each pair of stimulus types (c, continuous; i, intermittent) was calculated using correlation distance based on beta values of GLM analysis. (b) RDM result in the superior temporal gyrus (STG). (c) RDM result in SII. (d) Averaged RDM in each ROI

All voxels in anatomical ROIs (SI, STG, and SII) were extracted to investigate multi‐voxel activity patterns associated with stimulus types (Figure 3a–c). In addition, we averaged the index across stimulus types in each ROI. Results of paired *t* test showed significant higher dissimilarity in the SI compared with other brain areas (1,000 times bootstrap resample, *p* < .05), followed by STG, and SII (Figure 3d).

### 3.3 Comparison of stimulus types

**FIGURE 4:**
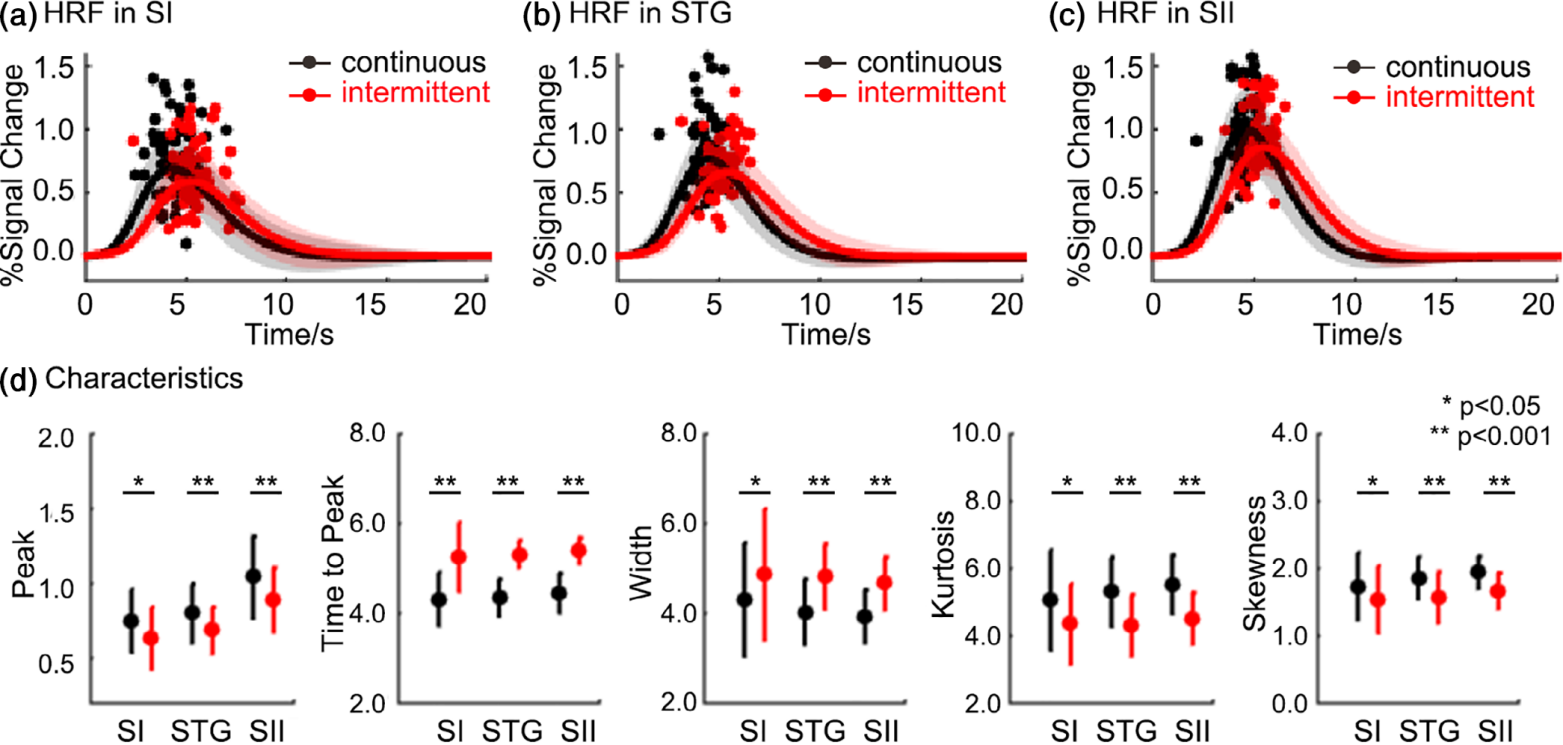
The HRF between the two patterns. The average HRF in (a) SI, (b) STG, and (c) SII. The dots are the individual peak times of HRFs from different subjects. (d) Estimated characteristics (peak, time to peak, width, kurtosis, and skewness) of the HRF. Black lines and dots represent continuous stimuli, while red lines and dots represent intermittent stimuli

The time series of these significant activated voxels were extracted for estimating the characteristics of the HRFs and were compared across stimulus types. All six free parameters of two gamma functions were summarized in Table S2. We did the same two‐way repeated measures ANOVA for each characteristic respectively. For the first factor pattern, results showed that there were significant main effects when comparing peak (SI: *p* = .002, F (1, 19) = 12.358; STG: *p* < .001, F (1, 19) = 20.630; SII: *p* < .001, F (1, 19) = 23.498), time to peak (SI: *p* < .001, F (1, 19) = 29.388; STG: *p* < .001, F (1, 19) = 80.475; SII: *p* < .001, F (1, 19) = 105.325), width (e.g., FWHM, SI: *p* = .002, F (1, 19) = 13.215; STG: *p* < .001, F (1, 19) = 38.636; SII: *p* < .001, F (1, 19) = 44.557), kurtosis (SI: *p* = .002, F (1, 19) = 12.812; STG: *p* < .001, F (1, 19) = 40.862; SII: *p* < .001, F (1, 19) = 63.672), skewness (SI: *p* = .015, F (1, 19) = 7.112; STG: *p* < .001, F (1, 19) = 21.916; SII: *p* < .001, F (1, 19) = 43.543). These results suggested that the continuous stimuli induced a stronger and earlier hemodynamic response than the intermittent stimuli. In addition, the HRF induced by continuous stimulus was significant narrower, especially in STG and SII (Figure 4d).

**FIGURE 5:**
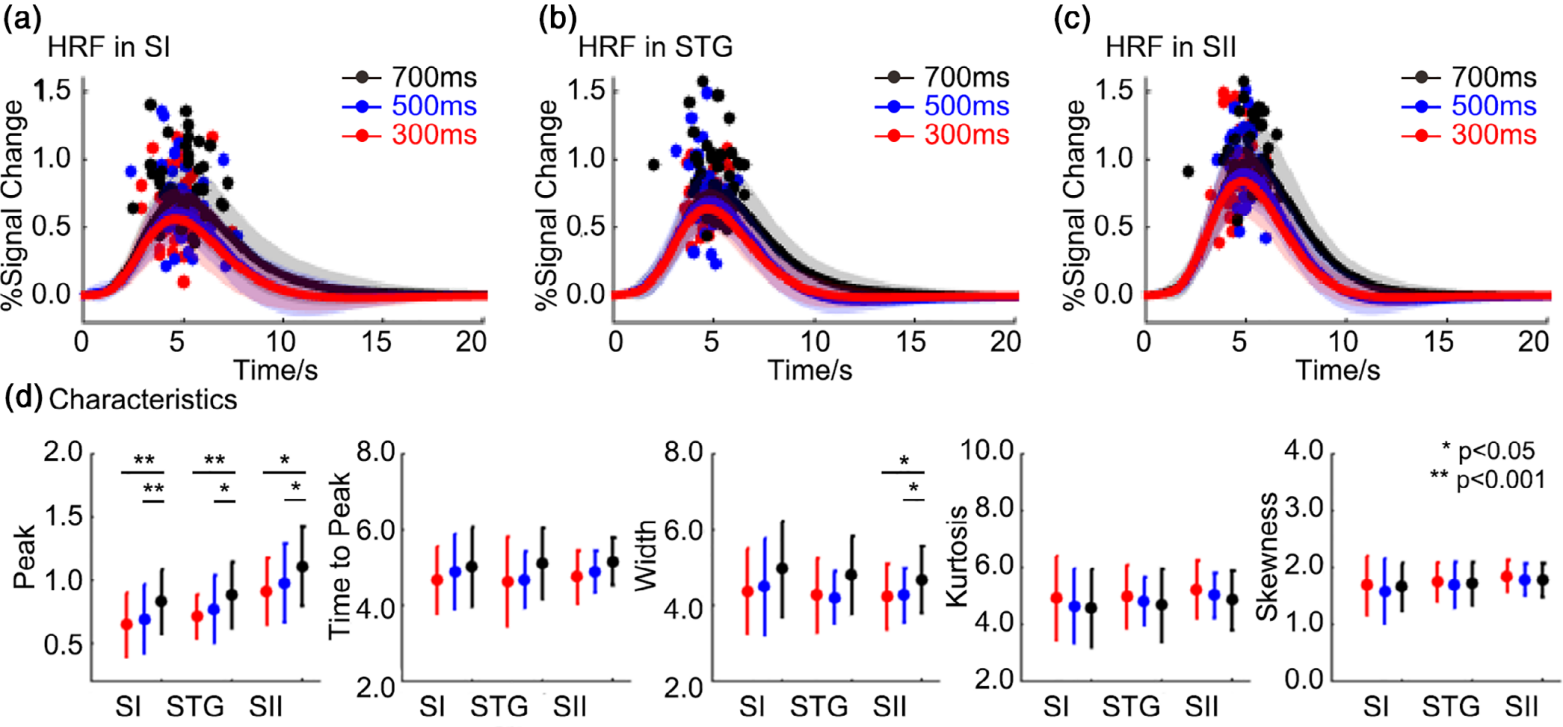
The HRFs across three durations. The average HRF in (a) SI, (b) STG, and (c) SII. The dots are the individual peak times of HRFs from different subjects. (d) Estimated characteristics (peak, time to peak, width, kurtosis, and skewness) of the HRF. Black lines and dots represent stimuli with the 700‐ms duration, blue lines, and dots represent the 500‐ms duration, and red lines and dots represent the 300‐ms duration

For the second factor duration, results of ANOVA revealed significant main effects on peak (SI: *p* < .001, F (2, 38) = 20.063; STG: *p* < .001, F (2, 38) = 12.674; SII: *p* < .001, F (2, 38) = 13.367) and width (SII: *p* = .005, F (2, 38) = 6.124). The paired comparison results showed that the magnitude of the HRF increased with increasing stimulus duration in all three ROIs (Figure 5d). In addition, the 700‐ms stimulus induced a longer width at half‐maximum of the HRF than the other stimulus in SII. Similar results were found using unsmoothed main dataset and verification data set (Figure S1–S4).

Furthermore, we designed two supplemental intermittent stimulus i5′ and i7′ and compared their induced HRF with i5 and i7. Each pair had the same duration but different number of pulses within 1 s period. All the characteristics were investigated, and paired‐sample *t* tests showed no significant differences between them. However, differences remained when compared with c5 and c7.

### 3.4 Nonlinear responses to brief stimulus

**FIGURE 6:**
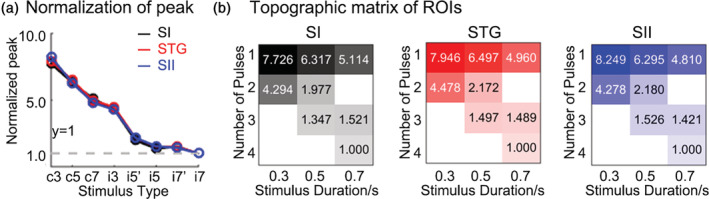
Normalized peak across different types of stimuli as an example. (a) Comparison across ROIs. (b) Topographic matrix in each ROI. The horizontal axis is the duration of the stimuli, and the vertical axis is the number of pulses within a period

To assess the nonlinearity of the hemodynamic response across stimulus durations and frequencies, we normalized the response by duration and number of pulse. Significant nonlinear responses were observed in each ROI for all the characteristics (the normalized indexes did not fall on the line y = 1, Figure 6a). The degree of nonlinearity was computed as the slope of the best‐fit straight line. Bootstrap 95% confidence interval (CI) of slope on peak was (−0.996, −0.724) in SI, (−0.929, −0.786) in STG, and (−0.899, −0.781) in SII, where zero is linear. For time to peak, CI of slope was (−1.185, −1.029) in SI, (−1.201, −1.083) in STG, and (−1.212, −1.065) in SII. Significant nonlinear were also observed in width (CI was [−1.234, −0.930] in SI, [−1.312, −1.036] in STG, and [−1.282, −1.048] in SII), kurtosis (CI was [−0.724, −0.513] in SI, [−0.647, −0.487] in STG, and [−0.606, −0.482] in SII), and skewness (CI was [−0.747, −0.570] in SI, [−0.747, −0.564] in STG, and [−0.690, −0.560] in SII).

In addition, topographic matrix of peak in each ROI as an example were displayed in Figure 6b. As shown in the matrix, it was clear that the normalized characteristic of the HRF decreased with increasing stimulus duration and number of pulses within a period. Once again, we could determine that stimuli with one pulse (continuous stimuli) could induce a stronger HRF than stimuli with more than one pulse.

## 4 DISCUSSION

To understand how signals are encoded to different types of tactile stimuli, we investigated the hemodynamic response induced by periodic tactile stimuli of the same intensity. We designed two patterns of stimuli with the same overall frequency (1 Hz) but different local temporal structures (continuous stimulus and intermittent stimulus with local frequencies of 2.5, 3.3, and 5 Hz). In addition, each pattern had three durations (300, 500, and 700 ms). First, we measured the spatial configuration, such as coordinates of peak activation and extent areas. Second, we estimated the HRF characteristics based on deconvolution of the sum of two gamma functions with six free parameters (Handwerker et al., 2004; Lindquist et al., 2009; Liu, Duffy, et al., 2017; Shan et al., 2014). Previous results showed that this function has low parameter bias for event‐related designs (Lindquist et al., 2009). The current results contribute to the evidence that the temporal structure of the stimuli is essential for vibrotactile perception.

Our results clearly demonstrated that continuous stimuli induced greater activation areas (Figure 2). In addition, continuous stimuli induced a significant stronger and narrower hemodynamic responses in all ROIs (Figure 4). Studies reported that the HRF variability could be affected by neural factors, such as the distribution and number of neurotransmitters and its receptors (Muthukumaraswamy, Evans, Edden, Wise, & Singh, 2012). HRFs induced by continuous stimuli had higher amplitudes because neurons fire more often. Different physiological profiles and response properties of mechanoreceptors related to continuous and intermittent stimuli may cause the variability of hemodynamic response. Research using Optacon found that intermittent stimulus evokes one spike per pulse in fast‐adapting type I and II tactile afferents without activating slow‐adapting type I afferents (Gardner & Palmer, 1989), which supports our results. Invasive studies in animals may ultimately be needed to determine the exact neural contributions to HRF. However, because the BOLD signal indirectly probed neuronal activity, differences of HRF between stimuli are more likely to reflect changes of vascular and metabolic events because the relatively slow timescale over neuronal activation (Uludag & Blinder, 2018). The hemodynamic response depends on combined changes in cerebral blood flow (CBF), cerebral blood volume (CBV), and cerebral metabolic rate of oxygen (CMRO2) (Buxton, 2012). Studies found the competition between CBF and the CMRO2 responses related to the task demand (Taylor et al., 2018). Although continuous and intermittent stimulus have similar temporal span, the on/off time of local pulses within one period are different. Stronger hemodynamic response of continuous stimuli perhaps indicates robust blood flow and subsequent oxygen metabolism during the brief stimulus. For intermittent stimuli, the mismatch of dynamics of CBF and CMRO2 may have caused more competition because there are more on/off within one period.

Furthermore, the amplitude of the HRF gradually increased as duration increased in SI, STG, and SII (Figure 5). This phenomenon has been demonstrated in auditory pitch perception, where longer inter‐pulse durations received higher weights than short ones (Carlyon, van Wieringen, Long, Deeks, & Wouters, 2002; Pressnitzer, Cheveigne, & Winter, 2004). A previous study reported that the number of suprathreshold voxels decreased during long durations of pressure stimulation, which was opposite to our results (Chung et al., 2015). However, the stimuli used in their study were up to 15 s, whereas, they were within 1 s in our study. We hypothesize that there may be a trend of growth followed by a decline at some time point, but assessing this is not within the scope of this study and could be explored in the future. This increase in amplitude may result from different total amounts of energy delivered by a stimulus. If the HRF is an idealized impulse response function that is identical across stimuli, this increase would be linear. However, when we normalized the response by duration and number of pulses within a cycle, the results showed a nonlinear response in all characteristics of hemodynamic response in all the ROIs. In other words, the peak amplitude and dynamics do not proportionately increase as the duration and number of pulses increase (Figure 6a). Shorter and fewer stimuli elicit proportionally larger responses. Nonlinearity has been observed in the visual cortex (Lewis et al., 2018; Yesilyurt, Ugurbil, & Uludag, 2008), auditory cortex (Soltysik, Peck, White, Crosson, & Briggs, 2004), and somatosensory cortex (Harrington & Downs, 2001; Nangini, MacIntosh, Tam, Staines, & Graham, 2005). Studies used a nonlinear neuronal model and have found it could not sufficiently explain the observed nonlinearity of HRF (Lewis et al., 2018). The current study does not provide sufficient data to discern possibly divergent responses between metabolic demands and blood flow increases (Puckett et al., 2014). Our research extended this nonlinear response to tactile related areas.

Although neural contribution and balance between vascular and metabolic demands cannot be completely disentangled in our results, we detected varied shapes of hemodynamic response induced by tactile stimuli with different temporal features. In addition, we included two supplemental stimuli i5′ and i7′ to further test whether the shape of the HRF could be modulated by number of pulses within one period (local frequency) at the same duration. The results showed that even if their local frequency was different, the HRF induced by them was similar and significant different from with the continuous stimulus. Consistent with previous results, the duration of a stimulus within a cycle was the key characteristic for discrimination frequency rather than the local frequency (Mackevicius et al., 2012). A behavioral study asked participants to make a judgment on perceived stimuli with varied temporal structure (Birznieks & Vickery, 2017). The stimuli had identical durations, but the local frequency was changed. The results revealed that the most essential feature determining tactile frequency was the duration between successive pulses. Several previous studies reported that early areas in the tactile circuits, such as the cuneate nucleus (Douglas, Ferrington, & Rowe, 1978) and VPL (Vazquez, Zainos, Alvarez, Salinas, & Romo, 2012), presented phase‐locked responses to the stimulus. However, this response was appreciably diminished in SI and almost disappeared in neurons of SII (Hernandez et al., 2010; Rossi‐Pool et al., 2016), which is consistent with results of the dissimilarity analysis, showing that the SI had significant higher level of dissimilarity across the stimulus types than the other brain areas (Figure 3). The pattern reflected the progressive flow of information. The faithful representation (high dissimilarity) was transformed to a more abstract representation (low dissimilarity), which seemed to extract and classify the features of the stimulus.

There are some limitations to our study. First, in this study, although participants could distinguish stimuli with different number of pulses, this does not contradict the fact that the stimuli induced similar HRFs. The participants may differentiate the stimuli by the different numbers of pulses. Previous studies pointed out that perceptual decisions seemed to build up across brain areas (Vazquez, Salinas, & Romo, 2013). Information encoded within limited areas did not entirely determine behavioral output. We need to further explore the differences in the brain network induced by different stimuli. Second, our study discussed only the positive peak of the HRF, but the poststimulus undershoot was also shown to be an important component of the HRF (Siero et al., 2015). We could explore variability in this component across stimulus types in future studies.

We conclude that this study characterizes the hemodynamic response induced by different types of tactile stimuli across tactile‐related areas. Firstly, continuous stimulus induced stronger and narrower hemodynamic response compared with intermittent stimulus. Secondly, the stimulation duration within a cycle may be a key component for distinguishing different stimuli. Nonlinearities of hemodynamic response are also found in tactile areas, which are related to stimulus duration and number of pulses. The competition between vascular and metabolic demand may be related to different tactile stimuli, and affects the variation and nonlinearity of HRF. We conclude that the temporal characteristics of tactile stimuli are an important component for distinguishing different stimuli. Future fMRI experimental designs and analyses should consider the varied HRF in different brain areas and induced by types of stimuli. In other words, if the temporal pattern of stimulation is not a contrast of interest for a study, it should be held constant across conditions.

## CONFLICT OF INTEREST

The authors declare that the research was conducted in the absence of any commercial or financial relationships that could be construed as potential conflicts of interest.

## AUTHOR CONTRIBUTIONS

Luyao Wang: Conceptualization, acquisition of data, analysis and interpretation the data, drafting the manuscript; Chunlin Li, Duanduan Chen, and Xiaoyu Lv: acquisition of data, analysis and interpretation the data; Ritsu Go, Jinglong Wu: revising the manuscript; Tianyi Yan: revising the manuscript.

## ETHICS STATEMENT

The protocol and data collection of the study were approved by the ethics committee of Capital Medical University in accordance with the declaration of Helsinki. All participants provided written informed consent.

## Supporting information


**Figure S1** The HRF between the two patterns based on unsmoothed data. The average HRF in (A) SI, (B) STG, and (C) SII. The dots are the individual peak times of HRFs from different subjects. (D) Estimated characteristics (peak, time to peak, width, kurtosis, and skewness) of the HRF. Black lines and dots represent continuous stimuli, while red lines and dots represent intermittent stimuli.
**Figure S2** The HRFs across three durations based on unsmoothed data. The average HRF in (A) SI, (B) STG, and (C) SII. The dots are the individual peak times of HRFs from different subjects. (D) Estimated characteristics (peak, time to peak, width, kurtosis, and skewness) of the HRF. Black lines and dots represent stimuli with the 700‐ms duration, blue lines and dots represent the 500‐ms duration, and red lines and dots represent the 300‐ms duration.
**Figure S3** The HRF between the two patterns based on unsmoothed verification data. The average HRF in (A) SI, (B) STG, and (C) SII. The dots are the individual peak times of HRFs from different subjects. (D) Estimated characteristics (peak, time to peak, width, kurtosis, and skewness) of the HRF. Black lines and dots represent continuous stimuli, while red lines and dots represent intermittent stimuli.
**Figure S4** The HRFs across three durations based on unsmoothed verification data. The average HRF in (A) SI, (B) STG, and (C) SII. The dots are the individual peak times of HRFs from different subjects. (D) Estimated characteristics (peak, time to peak, width, kurtosis, and skewness) of the HRF. Black lines and dots represent stimuli with the 700‐ms duration, blue lines and dots represent the 500‐ms duration, and red lines and dots represent the 300‐ms duration.
**Table S1.** Spatial coordinate of the peak activation voxel (MNI space)
**Table S2.** Parameters of two gamma function

## Data Availability

The data that support the findings of this study are available from the corresponding author upon reasonable request.
