# Supplementary material for: Hemodynamic response varies across tactile stimuli with different temporal structures
Source: Hum Brain Mapp. 2020 Nov 10;42(3):587–97. doi: 10.1002/hbm.25243 (PMC7814760; doi:10.1002/hbm.25243)
Supplement: Supplementary file 1 — Figure S1 The HRF between the two patterns based on unsmoothed data. The average HRF in (A) SI, (B) STG, and (C) SII. The dots are the individual peak times of HRFs from different subjects. (D) Estimated characteristics (peak, time to peak, width, kurtosis, and skewness) of the HRF. Black lines and dots represent continuous stimuli, while red lines and dots represent intermittent stimuli. Figure S2 The HRFs across three durations based on unsmoothed data. The average HRF in (A) SI, (B) STG, and (C) SII. The dots are the individual peak times of HRFs from different subjects. (D) Estimated characteristics (peak, time to peak, width, kurtosis, and skewness) of the HRF. Black lines and dots represent stimuli with the 700‐ms duration, blue lines and dots represent the 500‐ms duration, and red lines and dots represent the 300‐ms duration. Figure S3 The HRF between the two patterns based on unsmoothed verification data. The average HRF in (A) SI, (B) STG, and (C) SII. The dots are the individual peak times of HRFs from different subjects. (D) Estimated characteristics (peak, time to peak, width, kurtosis, and skewness) of the HRF. Black lines and dots represent continuous stimuli, while red lines and dots represent intermittent stimuli. Figure S4 The HRFs across three durations based on unsmoothed verification data. The average HRF in (A) SI, (B) STG, and (C) SII. The dots are the individual peak times of HRFs from different subjects. (D) Estimated characteristics (peak, time to peak, width, kurtosis, and skewness) of the HRF. Black lines and dots represent stimuli with the 700‐ms duration, blue lines and dots represent the 500‐ms duration, and red lines and dots represent the 300‐ms duration. Table S1. Spatial coordinate of the peak activation voxel (MNI space) Table S2. Parameters of two gamma function [file HBM-42-587-s001.docx]

**Supplementary Materials for**

**Hemodynamic Response Varies across Tactile Stimuli with Different Temporal Structures**

Luyao Wang^1,4^, Chunlin Li^2^, Duanduan Chen^3^, Xiaoyu Lv^1,4^, Ritsu Go^1,4^, Jinglong Wu^1,4,5^, Tianyi Yan^3,4*^

^1^ School of Mechatronical Engineering, Beijing Institute of Technology, Beijing, China

^2^ School of Biomedical Engineering, Capital Medical University, Beijing, China.

^3^ School of Life Science, Beijing Institute of Technology, Beijing, China

^4^ Beijing Advanced Innovation Center for Intelligent Robots and Systems, Beijing Institute of Technology, Beijing, China

^5^ Graduate School of Interdisciplinary Science and Engineering in Health Systems, Okayama University, Okayama, Japan

*******Correspondence:**

**Tianyi Yan**: [yantianyi@bit.edu.cn](mailto:yantianyi@bit.edu.cn)

School of Life Science, Beijing Institute of Technology, Beijing, 5 South Zhongguancun Street, Haidian District, Beijing 100081, China.

**This file includes:**

Figures S1 to S4

Tables S1 to S2

**Supplementary Figures**

**
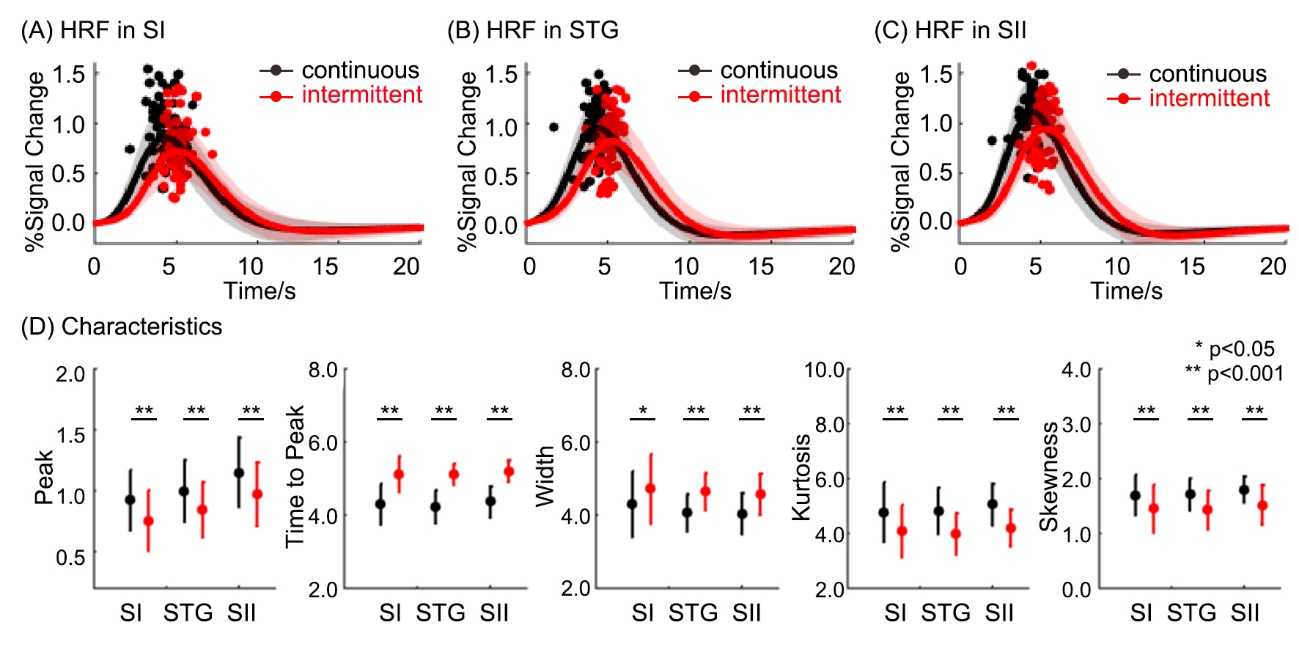
**

**Figure S1** The HRF between the two patterns based on unsmoothed data. The average HRF in (A) SI, (B) STG, and (C) SII. The dots are the individual peak times of HRFs from different subjects. (D) Estimated characteristics (peak, time to peak, width, kurtosis, and skewness) of the HRF. Black lines and dots represent continuous stimuli, while red lines and dots represent intermittent stimuli.

**
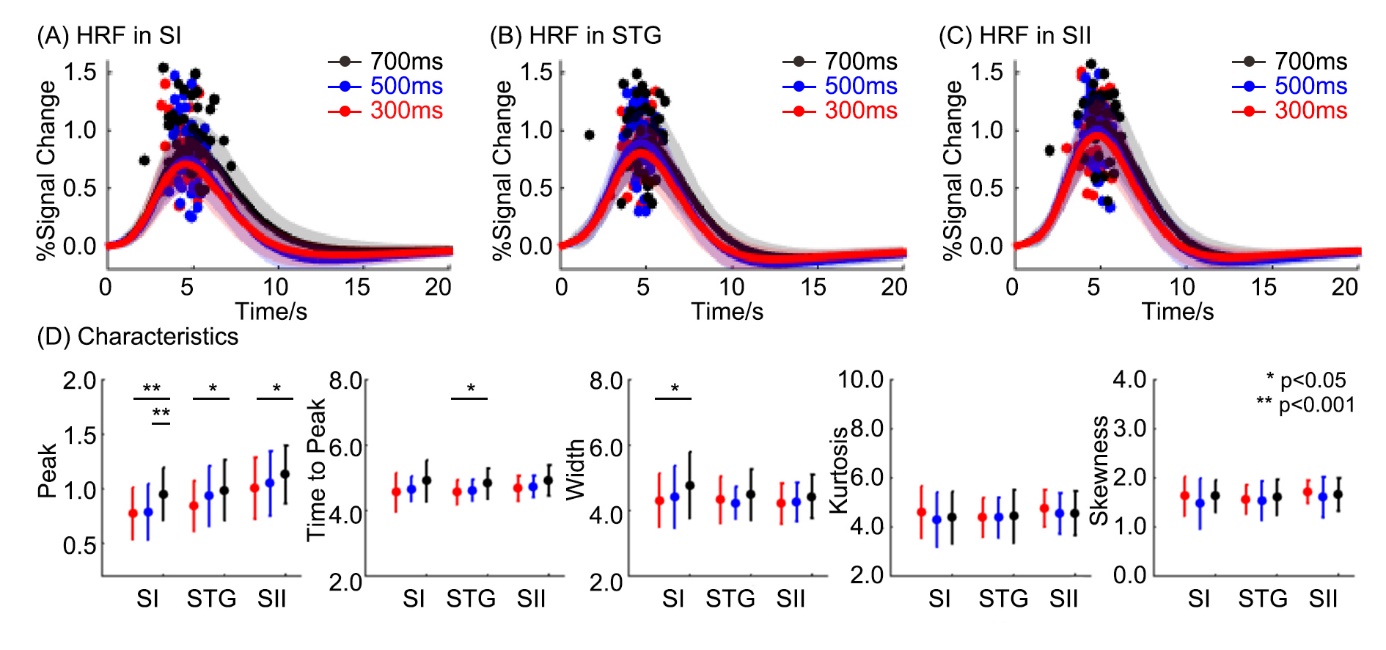
**

**Figure S2** The HRFs across three durations based on unsmoothed data. The average HRF in (A) SI, (B) STG, and (C) SII. The dots are the individual peak times of HRFs from different subjects. (D) Estimated characteristics (peak, time to peak, width, kurtosis, and skewness) of the HRF. Black lines and dots represent stimuli with the 700-ms duration, blue lines and dots represent the 500-ms duration, and red lines and dots represent the 300-ms duration.

**
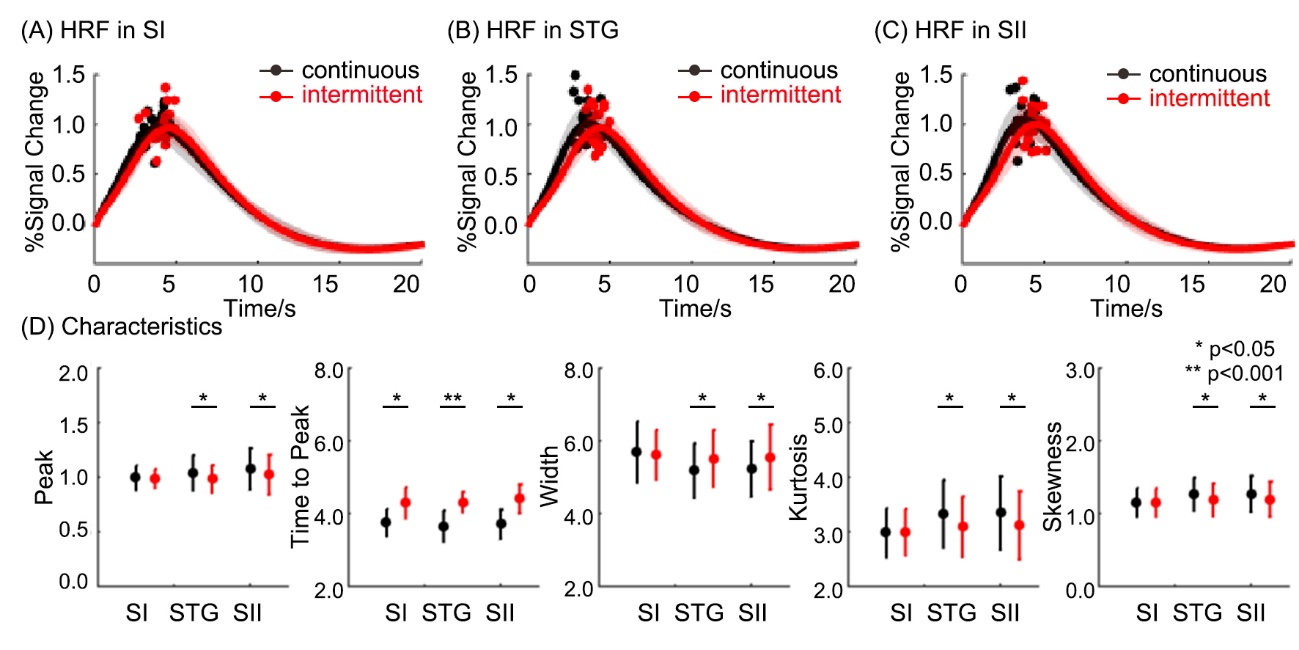
**

**Figure S3** The HRF between the two patterns based on unsmoothed verification data. The average HRF in (A) SI, (B) STG, and (C) SII. The dots are the individual peak times of HRFs from different subjects. (D) Estimated characteristics (peak, time to peak, width, kurtosis, and skewness) of the HRF. Black lines and dots represent continuous stimuli, while red lines and dots represent intermittent stimuli.

**
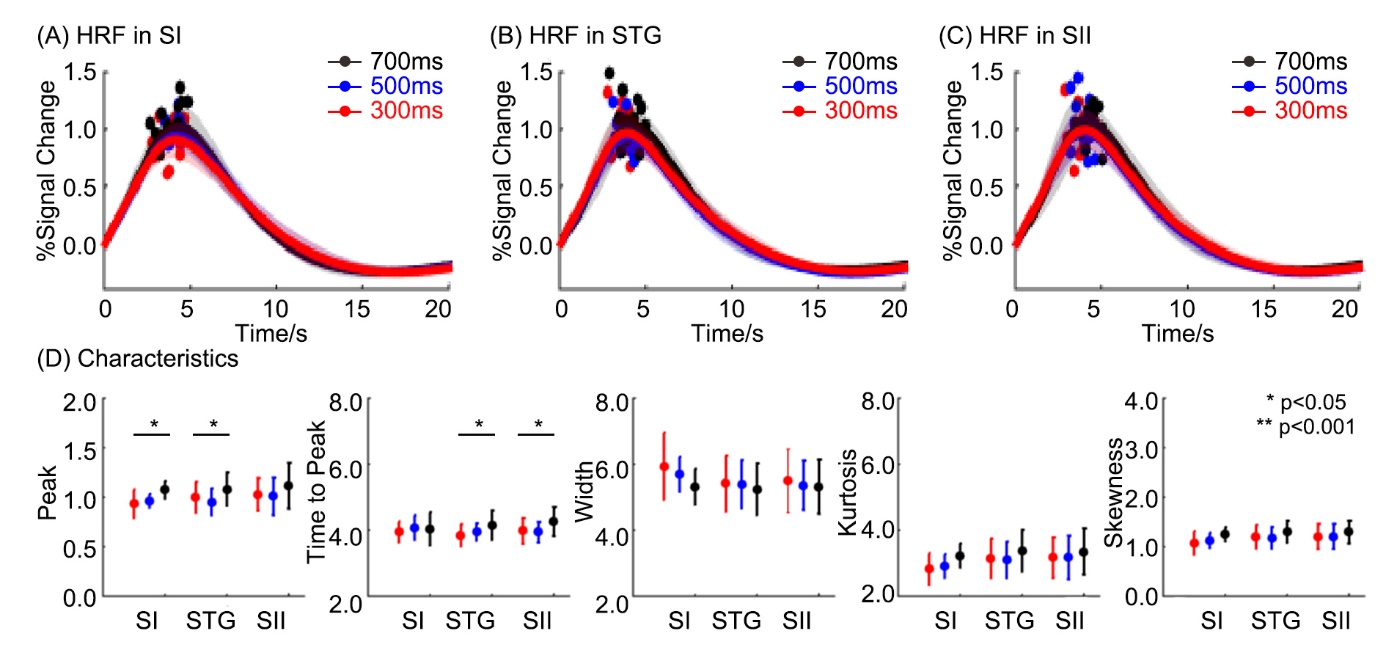
**

**Figure S4** The HRFs across three durations based on unsmoothed verification data. The average HRF in (A) SI, (B) STG, and (C) SII. The dots are the individual peak times of HRFs from different subjects. (D) Estimated characteristics (peak, time to peak, width, kurtosis, and skewness) of the HRF. Black lines and dots represent stimuli with the 700-ms duration, blue lines and dots represent the 500-ms duration, and red lines and dots represent the 300-ms duration.

**Supplementary Tables**

**Table S1. Spatial coordinate of the peak activation voxel (MNI space)**

|  | **SI** | | | **STG** | | | **SII** | | |
| --- | --- | --- | --- | --- | --- | --- | --- | --- | --- |
|  | **x** | **y** | **z** | **x** | **y** | **z** | **x** | **y** | **z** |
| **c3** | -48 | -22 | 46 | -42 | -34 | 10 | -54 | -28 | 20 |
| **c5** | -48 | -22 | 46 | -44 | -34 | 10 | -50 | -28 | 18 |
| **c7** | -48 | -22 | 46 | -44 | -34 | 10 | -50 | -30 | 14 |
| **i3** | -46 | -22 | 48 | -42 | -34 | 10 | -50 | -28 | 18 |
| **i5** | -46 | -22 | 48 | -44 | -34 | 10 | -52 | -28 | 16 |
| **i7** | -44 | -22 | 52 | -40 | -34 | 12 | -54 | -18 | 16 |
| **i5’** | -66 | -22 | 22 | -42 | -36 | 12 | -50 | -26 | 18 |
| **i7’** | -46 | -22 | 48 | -64 | -38 | 14 | -50 | -32 | 16 |

**Table S2. Parameters of two gamma function**

|  |  | **c3** | **c5** | **c7** | **i3** | **i5** | **i7** | **i5’** | **i7’** |
| --- | --- | --- | --- | --- | --- | --- | --- | --- | --- |
| **Smoothed** | **SI** |  |  |  |  |  |  |  |  |
|  | A | 4.556 | 5.563 | 6.354 | 6.537 | 4.874 | 6.232 | 4.914 | 6.235 |
|  | Alpha1 | 7.590 | 7.240 | 7.354 | 7.933 | 8.307 | 7.915 | 8.223 | 7.515 |
|  | Beta1 | 1.256 | 1.250 | 1.240 | 1.116 | 1.157 | 1.117 | 1.186 | 1.106 |
|  | C | 2.574 | 1.719 | 1.811 | 1.930 | 2.747 | 1.694 | 2.870 | 1.731 |
|  | Alpha2 | 15.319 | 16.052 | 15.782 | 15.852 | 14.796 | 15.852 | 14.702 | 15.888 |
|  | Beta2 | 0.905 | 0.856 | 0.820 | 0.855 | 0.983 | 0.910 | 0.951 | 0.884 |
|  | **STG** |  |  |  |  |  |  |  |  |
|  | A | 5.369 | 6.353 | 6.889 | 7.172 | 5.359 | 7.281 | 5.345 | 7.040 |
|  | Alpha1 | 8.219 | 7.587 | 8.185 | 7.932 | 8.400 | 8.101 | 8.275 | 7.637 |
|  | Beta1 | 1.293 | 1.226 | 1.290 | 1.058 | 1.190 | 1.057 | 1.170 | 1.046 |
|  | C | 2.781 | 1.861 | 2.056 | 1.952 | 3.004 | 2.057 | 2.798 | 1.896 |
|  | Alpha2 | 14.812 | 16.132 | 15.971 | 15.932 | 14.793 | 15.758 | 15.053 | 15.832 |
|  | Beta2 | 0.892 | 0.858 | 0.819 | 0.848 | 0.957 | 0.918 | 0.955 | 0.926 |
|  | **SII** |  |  |  |  |  |  |  |  |
|  | A | 5.294 | 6.286 | 6.655 | 6.766 | 5.565 | 7.340 | 5.821 | 7.270 |
|  | Alpha1 | 8.363 | 7.636 | 8.250 | 8.015 | 8.669 | 8.163 | 8.531 | 7.830 |
|  | Beta1 | 1.478 | 1.376 | 1.448 | 1.198 | 1.325 | 1.170 | 1.262 | 1.160 |
|  | C | 2.628 | 1.838 | 1.862 | 1.868 | 2.869 | 1.974 | 2.986 | 1.847 |
|  | Alpha2 | 14.208 | 15.718 | 15.529 | 15.406 | 14.162 | 14.981 | 14.577 | 15.403 |
|  | Beta2 | 0.912 | 0.868 | 0.829 | 0.865 | 0.970 | 0.945 | 0.958 | 0.934 |
| **UnSmoothed** | **SI** |  |  |  |  |  |  |  |  |
|  | A | 4.465 | 5.534 | 6.055 | 5.192 | 4.372 | 6.188 | 4.190 | 6.077 |
|  | Alpha1 | 7.099 | 6.869 | 6.901 | 7.330 | 7.667 | 7.466 | 7.570 | 7.321 |
|  | Beta1 | 1.420 | 1.320 | 1.339 | 1.279 | 1.315 | 1.202 | 1.337 | 1.205 |
|  | C | 3.061 | 2.587 | 2.387 | 2.638 | 3.793 | 2.736 | 3.606 | 2.864 |
|  | Alpha2 | 15.633 | 16.115 | 16.024 | 15.703 | 15.275 | 15.766 | 15.412 | 15.880 |
|  | Beta2 | 0.997 | 0.972 | 0.953 | 0.969 | 1.024 | 0.988 | 1.006 | 0.977 |
|  | **STG** |  |  |  |  |  |  |  |  |
|  | A | 4.857 | 6.058 | 6.246 | 6.040 | 5.376 | 6.388 | 5.001 | 6.675 |
|  | Alpha1 | 7.224 | 6.997 | 7.201 | 7.240 | 7.753 | 7.719 | 7.599 | 7.496 |
|  | Beta1 | 1.436 | 1.361 | 1.421 | 1.266 | 1.345 | 1.235 | 1.330 | 1.226 |
|  | C | 3.845 | 3.090 | 3.237 | 3.067 | 4.367 | 3.683 | 4.100 | 3.629 |
|  | Alpha2 | 15.168 | 16.059 | 15.835 | 15.869 | 15.140 | 15.554 | 15.357 | 15.630 |
|  | Beta2 | 0.883 | 0.858 | 1.181 | 1.102 | 0.810 | 1.114 | 0.687 | 1.110 |
|  | **SII** |  |  |  |  |  |  |  |  |
|  | A | 5.472 | 6.690 | 6.934 | 6.837 | 5.912 | 7.022 | 5.494 | 7.317 |
|  | Alpha1 | 7.431 | 7.150 | 7.376 | 7.469 | 7.787 | 7.778 | 7.761 | 7.626 |
|  | Beta1 | 1.457 | 1.368 | 1.421 | 1.272 | 1.322 | 1.232 | 1.330 | 1.233 |
|  | C | 3.599 | 2.870 | 3.132 | 3.057 | 4.241 | 3.693 | 4.151 | 3.621 |
|  | Alpha2 | 14.980 | 15.599 | 15.510 | 15.506 | 14.989 | 15.277 | 15.105 | 15.371 |
|  | Beta2 | 1.002 | 0.976 | 0.979 | 0.992 | 1.034 | 1.026 | 1.033 | 1.034 |
